# Hepatitis E Virus Infection without Reactivation in Solid-Organ Transplant Recipients, France

**DOI:** 10.3201/eid1701.100527

**Published:** 2011-01

**Authors:** Florence Legrand-Abravanel, Nassim Kamar, Karine Sandres-Saune, Sebastien Lhomme, Jean-Michel Mansuy, Fabrice Muscari, Federico Sallusto, Lionel Rostaing, Jacques Izopet

**Affiliations:** Author affiliations: Institut National de la Santé et de la Recherche Médicale, Toulouse, France (F. Legrand-Abravanel, N. Kamar, K. Sandres-Saune, S. Lhomme, L. Rostaing, J. Izopet);; Centre Hospitalier Universitaire Toulouse, Toulouse (F. Legrand-Abravanel, N. Kamar, K. Sandres-Saune, S. Lhomme, J.-M. Mansuy, F. Muscari, F. Sallusto, L. Rostaing, J. Izopet)

**Keywords:** Hepatitis E virus, viruses, incidence, reactivation, solid-organ transplant recipients, France, research

## MEDSCAPE CME

Medscape, LLC is pleased to provide online continuing medical education (CME) for this journal article, allowing clinicians the opportunity to earn CME credit.

This activity has been planned and implemented in accordance with the Essential Areas and policies of the Accreditation Council for Continuing Medical Education through the joint sponsorship of Medscape, LLC and Emerging Infectious Diseases. Medscape, LLC is accredited by the ACCME to provide continuing medical education for physicians.

Medscape, LLC designates this Journal-based CME for a maximum of 1 *AMA PRA Category 1 Credit(s)*^TM^. Physicians should claim only the credit commensurate with their participation in the activity.

All other clinicians completing this activity will be issued a certificate of participation. To participate in this journal CME activity: (1) review the learning objectives and author disclosures; (2) study the education content; (3) take the post-test and/or complete the evaluation at www.medscapecme.com/journal/eid; (4) view/print certificate.

**Release date: December 22, 2010; Expiration date: December 22, 2011**

## Learning Objectives

Upon completion of this activity, participants will be able to:

- Describe factors associated with higher risk for HEV seroconversion and development of chronic disease among liver and kidney transplant patients.

## MEDSCAPE CME Editor

**Thomas J. Gryczan, MS, **Technical Writer-Editor,* Emerging Infectious Diseases. Disclosure: Thomas J. Gryczan, MS, has disclosed no relevant financial relationships.*

## MEDSCAPE CME AUTHOR

**Desiree Lie, MD, MSEd, **Clinical Professor; Director of Research and Faculty Development, Department of Family Medicine, University of California, Irvine at Orange. *Disclosure: Désirée Lie, MD, MSEd, has disclosed the following relevant financial relationship: served as a nonproduct speaker for "Topics in Health" for Merck Speaker Services.*

## AUTHORS

*Disclosures:* ***Florence Legrand-Abravanel, PharmD, PhD; Nassim Kamar, MD, PhD; Karine Sandres-Saune, PharmD, PhD; Sebastien Lhomme, PharmD; Jean-Michel Mansuy, MD; Fabrice Muscari, MD, PhD; Federico Sallusto, MD; Lionel Rostaing, MD, PhD;*** *and* ***Jacques Izopet, PharmD, PhD,*** *have disclosed no relevant financial relationships.*
