# Supplementary material for: Hepatitis E Virus Infection without Reactivation in Solid-Organ Transplant Recipients, France
Source: Emerg Infect Dis. 2011 Jan;17(1):30–7. doi: 10.3201/eid1701.100527 (PMC3298369; doi:10.3201/eid1701.100527)
Supplement: Related texts

# Research article: Hepatitis E Virus Infection without Reactivation in Solid-Organ Transplant Recipients, France

**DOI:** 10.3201/eid1701.100527

**Authors:** Florence Legrand-Abravanel, Nassim Kamar, Karine Sandres-Saune, Sebastien Lhomme, Jean-Michel Mansuy, Fabrice Muscari, Federico Sallusto, Lionel Rostaing, Jacques Izopet

**Affiliations:** Author affiliations: Institut National de la Santé et de la Recherche Médicale, Toulouse, France (F. Legrand-Abravanel, N. Kamar, K. Sandres-Saune, S. Lhomme, L. Rostaing, J. Izopet);; Centre Hospitalier Universitaire Toulouse, Toulouse (F. Legrand-Abravanel, N. Kamar, K. Sandres-Saune, S. Lhomme, J.-M. Mansuy, F. Muscari, F. Sallusto, L. Rostaing, J. Izopet)

## Abstract

Infections with hepatitis E virus (HEV) in solid-organ transplant recipients can lead to chronic hepatitis. However, the incidence of de novo HEV infections after transplantation and risk for reactivation in patients with antibodies against HEV before transplantation are unknown. Pretransplant prevalence of these antibodies in 700 solid-organ transplant recipients at Toulouse University Hospital in France was 14.1%. We found no HEV reactivation among patients with antibodies against HEV at the first annual checkup or by measuring liver enzyme activities and HEV RNA. In contrast, we found 34 locally acquired HEV infections among patients with no antibodies against HEV, 47% of whom had a chronic infection, resulting in an incidence of 3.2/100 person-years. Independent risk factors for HEV infection were an age <52 years at transplantation and receiving a liver transplant. Effective prophylactic measures that include those for potential zoonotic infections should reduce the risk for HEV transmission in this population.

Large epidemics of hepatitis E have occurred in Asia, Africa, and Latin America where sanitation is suboptimal (*1*). The numbers of non–travel-associated hepatitis E virus (HEV) infections have also increased in industrialized countries in recent years, and these infections are now considered an emerging infectious disease in Western countries (*2*). HEV belongs to the family *Hepeviridae*. At least 4 major genotypes of HEV have been recognized. Genotypes 1 and 2 are restricted to humans and associated with epidemics in developing countries, and genotypes 3 and 4 are zoonotic and infect humans and several other animals in developing and industrialized countries (*3*). The discovery of animal strains of HEV in pigs, wild boars, deer, and rodents and the finding of other animal species with antibodies against HEV has broadened the host range and diversity of HEV (*3*).

Although HEV is transmitted mainly by the fecal–oral route in developing countries, there are other routes in industrialized countries. These routes include contact with animal reservoirs (*4**,**5*) and consumption of undercooked pig, deer, or wild boar meat (*6**–**8*). Nosocomial infection and transfusion-transmitted infection have also been reported (*9**–**11*). HEV infection generally has a self-limiting symptomatic course or an asymptomatic course that includes acute hepatitis. Fulminant hepatitis may occur in pregnant women and persons with underlying liver disease (*1**,**12**,**13*). HEV can lead to chronic infection in solid-organ transplant (SOT) patients (*14**–**16*), in patients who have had chemotherapy (*11**,**17**,**18*), and in patients infected with HIV (*19**,**20*). HEV infection can evolve to chronic hepatitis in up to 60% of infected SOT patients (*16*).

Reactivation of an HEV infection was reported recently in a patient in Germany who had acute lymphoblastic leukemia after allogeneic stem cell transplantation (*21*). This reactivation was diagnosed as acute limited hepatitis E before stem cell transplantation. HEV viremia reappeared 14 weeks after stem cell transplantation with increased aminotransferase activity. Analysis of virus RNA sequences showed reactivation of endogenous HEV genotype 3, which indicated that the virus had persisted despite the recovery of the patient from acute hepatitis E (*21*).

Southwestern France is characterized by a high prevalence of antibodies against HEV in blood donors (*22*), and several cases of HEV infections have been reported in immunocompetent and immunocompromised patients (*23**,**24*). However, the incidence and risk for reactivation of HEV infection in SOT patients in this area are unknown. To identify these factors, we examined a large cohort of immunocompromised patients at Toulouse University Hospital, France, during January 2004–December 2009 to determine the prevalence of antibodies against HEV in SOT recipients before transplantation, the frequency of HEV reactivation, the incidence of HEV infections relative to the organ transplanted, and the risk for HEV transmission from the organ donor or from a blood transfusion.

## Patients and Methods

### Patients

A total of 808 adult patients underwent transplantation of a kidney or liver during January 2004–December 2008 at Toulouse University Hospital. All adult patients for whom a blood sample obtained on the day of transplantation was available were included in the study (n = 700, 529 kidney transplant recipients and 171 liver transplant recipients). Serum samples obtained on the day of transplantation and during annual checkups until December 2009 were tested for immunoglobulin (Ig) M and IgG against HEV. Positive samples were tested for HEV RNA.

Patients with pretransplant antibodies against HEV were investigated for HEV reactivation by testing for HEV RNA at the first annual checkup and for increased liver enzyme activities during follow-up. Patients without pretransplant antibodies who underwent IgG seroconversion and patients who had unexplained acutely increased liver enzyme activities during follow-up were also tested for HEV RNA. Patients whose test results were antibody negative before transplantation were monitored from the date of transplantation until the date of IgG seroconversion or HEV infection, death, last health care encounter, or study end (December 31, 2009).

Frozen serum samples from donors (obtained at Toulouse University Hospital) who had donated an organ to an HEV-infected recipient were also tested for IgG and IgM against HEV and HEV RNA to determine whether transmission had occurred by organ transplantation. Similarly, frozen serum samples from donors who gave blood to an HEV-infected recipient were also tested for IgG and IgM against HEV and HEV RNA to determine whether HEV infection had occurred by blood transfusion.

### Case Definitions

Patients who had IgG, IgM, or both against HEV but did not have HEV RNA were considered uninfected. Patients who were negative for antibodies against HEV at transplantation and who showed HEV IgG seroconversion during follow-up were considered to have a de novo HEV infection. Patients who were positive for HEV RNA were also considered to have an HEV infection that was independent of IgG or IgM against HEV.

### Laboratory Investigation for HEV Infection

We detected IgM and IgG against HEV by using EIAgen HEV IgG and EIAgen HEV IgM Kits (Adaltis Ingen, Paris, France). Ratios of antibodies against HEV were sample optical densities/cutoff optical densities. A result was considered positive if the sample ratio was >1. We used real-time PCRs and a hydrolysis probe to detect HEV RNA (*24**,**25*). Virus genotype was determined by sequencing a 189-nt fragment within the open reading frame 2 gene (*26*). Phylogenetic analyses were performed by using genotype information of reference sequences based on the HEV classification proposed by Lu et al. (*27*).

### Statistical Analysis

Analyses were performed by using Stata version 9.2 (StataCorp LP, College Station, TX, USA). The χ^2^ test or Fisher exact test was used to compare proportions. Student *t* test was used to compare continuous variables. A p value <0.05 was considered significant.

Demographic and clinical factors associated with pretransplant antibodies against HEV were evaluated by using univariate analyses. We analyzed sex, age at transplantation (<52 years or >52 years), place of residence on the basis of community size (<20,000 or >20,000 inhabitants), type and cause of transplantation, and immunosuppressive therapy at discharge (each therapy or triple and double immunosuppressive regimens). Variables with a p value <0.10 by univariate analysis were entered into a multivariate, backward, stepwise logistic regression analysis to identify variables independently associated with pretransplant HEV seroprevalence.

Incidence of HEV infection and 95% confidence intervals (CIs) among SOT patients negative for antibodies against HEV was calculated. For the purpose of estimating the date of HEV infection for patients with HEV IgG seroconversion, we used the date midway between the last available seronegative test result and the first available seropostive test result. Demographic and clinical risk factors were entered into a Poisson regression to assess factors associated with de novo HEV infection. Risks are expressed as incidence rate ratios and their 95% CIs.

## Results

### Pretransplant HEV Seroprevalence

Blood samples from 700 SOT recipients were screened on the day of transplantation. Median age of patients at transplantation was 52 years (range 18–79 years); 459 (65.6%) of the patients were men (Table 1). No demographic or clinical factors were associated with pretransplant antibodies against HEV. IgG, IgM, or both antibodies against HEV were found in 99 patients (14.1%): 77 of the 529 kidney-transplant patients (14.5%) and 22 of the 171 liver-transplant patients (12.9%) (Figure 1). Serum from 17 patients (2.4%) contained HEV IgM (7 of these patients were also IgG positive). None of the 99 patients who had antibodies against HEV had HEV RNA. Pretransplant seroprevalence varied according to year of transplantation (range 8.7%–16.3%), although these variations were not significant.

**Table 1:** Characteristics of 700 solid-organ transplant recipients and seroprevalence of antibodies against HEV before transplant, France, January 2004–December 2008*

Characteristic	No. patients	HEV IgG positive, no. (%)	HEV IgM positive, no. (%)
Sex			
M	459	57 (12.4)	8 (1.7)
F	241	32 (13.2)	9 (3.7)
Age at transplantation, y			
<52	347	43 (12.4)	8 (2.3)
>52	363	46 (13.0)	9 (2.5)
Transplant type			
Liver	171	21 (12.3)	4 (2.3)
Kidney	529	68 (12.8)	13 (2.4)
Cause of kidney transplantation			
Glomerulonephritis	188	27 (14.3)	4 (2.1)
Genetic nephritis	102	15 (14.7)	1 (0.9)
Pyelonephritis or interstitial nephritis	92	7 (7.6)	2 (2.1)
Nephroangiosclerosis	82	9 (10.9)	2 (2.4)
Other	57	10 (17.5)	4 (7)
Cause of liver transplantation			
Alcoholic cirrhosis	67	6 (8.9)	0
Hepatitis B or C	60	7 (11.6)	2 (3.3)
Autoimmune cirrhosis	9	2 (22)	1 (11)
Other	35	6 (17.1)	1 (2.8)
Hepatocarcinoma (yes/no)	64/107	9 (14)	2 (3.1)
Induction treatment (yes/no)	501/199	73 (14.5)	14 (2.8)
Interleukin-2 receptor blockers	292	40 (7.3)	4 (1.3)
Rabbit antithymocyte globulins	160	25 (15.6)	8 (5)
Immunosuppressive therapy at discharge			
Belatacept	49	8 (16.3)	2 (16.3)
Cyclosporine A	182	27 (15.3)	1 (0.5)
Tacrolimus	442	52 (11.7)	14 (3.1)
Steroids	657	85 (12.9)	17 (2.5)
Mycophenolate	593	74 (12.5)	15 (2.6)
Azathioprine	2	0	0

*HEV, hepatitis E virus; Ig, immunoglobulin.

**Figure 1:** Hepatitis E virus (HEV) markers in 700 solid-organ transplant recipients, France, January 2004–December 2008. Ig, immunoglobulin.

### HEV Reactivation in Pretransplant Seropositive Patients

We determined HEV reactivation in 99 pretransplant HEV-seropositive patients whose blood samples obtained at the first annual checkup were negative for HEV RNA (Figure 1). All blood samples were negative for HEV RNA. We also checked clinical records to identify any episodes of hepatic cytolysis. Liver enzyme activities of 38 patients were not increased during follow-up, but there were 80 episodes of hepatic cytolysis in the other 61 patients. None of the blood samples obtained during episodes of hepatic cytolysis was positive for HEV RNA.

The 89 pretransplant patients who were positive for HEV IgG included 84 patients who underwent a serologic test after a median of 24 months (interquartile range [IQR] 14–36 months). Thirty-two (35.9%) patients who were positive for HEV IgG became negative. Disappearance of antibodies against HEV was not associated with type of immunosuppressive therapy at discharge. The 17 pretransplant HEV IgM–positive patients included 16 patients who underwent a serologic test after a median of 16 months (IQR 12–23 months). Five of the 16 patients were still positive for HEV IgM but negative for HEV RNA at this time.

### Incidence of HEV Infection

The 601 pretransplant HEV IgG– and HEV IgM–negative patients included 452 kidney transplant recipients and 149 liver transplant recipients. Of these patients, 76 kidney transplant recipients and 26 liver transplant recipients were lost to follow-up. Median follow-up time was 22 months (IQR 12–36 months); total follow-up time was 1,064 person-years after transplantation. A total of 34 HEV infections were identified: 22 were in kidney transplant recipients and 12 were in liver transplant recipients (Figure 1). Median interval between transplantation and de novo HEV infections was 15 months (IQR 6–30 months).

We diagnosed HEV infections in 20 patients with increased liver enzyme activities by detecting HEV RNA; HEV infections in 14 other patients were diagnosed by detection of HEV IgG during posttransplant follow-up. Nine of these 14 patients also had HEV IgM (Figure 2). Investigation of clinical records led to detection of HEV RNA–positive samples in 4 of these patients by retrospective analysis of frozen stored blood samples. HEV RNA was not detected in the other 10 patients because no samples were available when alanine aminotransferase levels peaked.

**Figure 2:** Diagnosis and characteristics of 34 patients with hepatitis E virus (HEV) infection, France, January 2004–December 2008. Ig, immunoglobulin; NA, not available.

Incidence of HEV infection was 3.2 cases/100 person-years (95% CI 2.06–4.13 cases). The incidence in liver transplant recipients (4.8 cases/100 person-years; 95% CI 2.2–7.4 cases) tended to be higher than in kidney transplant recipients (2.7 cases/100 person-years; 95% CI 1.52–3.68 cases; p = 0.09). Incidence rates for each year after transplantation (Table 2) ranged from 0.9 to 4.3 cases/100 person-years in kidney transplant recipients and from 0 to 7.1 cases/100 person-years in liver transplant recipients, but the differences were not significant. The incidence according to calendar year of transplantation varied from 1.2 to 4.3 cases/100 person-years in kidney transplant recipients and from 2.5 to 9.1 cases/100 person-years in liver transplant recipients. Incidence of HEV infection according to calendar year of transplantation did not differ significantly over the 5-year study period.

**Table 2:** Incidence of HEV per year after transplantation in 601 solid-organ transplant recipients, France, January 2004–December 2008*

Patient group	Incidence, cases/100 person-years (95% confidence interval)					p value
	Global	First year	Second year	Third year	Fourth year	
All	3.2 (2.06–4.13)	3.5 (1.9–5.1)	1.1 (0.03–2.19)	3.7 (1.09–6.1)	3.0 (0.1–7.52)	0.08
Kidney transplant	2.7 (1.52–3.68)	2.4 (0.8–3.9)	0.9 (0.01–4.4)	3.3 (0.47–6.120)	4.3 (1.28–11.48)	0.18
Liver transplant	4.8 (2.2–7.4)	7.12 (2.63–11.62)	1.6 (0.04–7.60)	5.1 (0.52–14.54)	0 (0–10)	0.24

*HEV, hepatitis E virus.

### HEV Transmission by Organ Transplant or Blood Transfusion

We studied the risk for HEV transmission by organ transplant by identifying virologic markers in 15 persons who provided organs for 15 HEV-infected SOT recipients (44%). Eight recipients were infected with HEV in the first year posttransplantation, 3 were HEV positive 1–2 years posttransplantation, 3 were HEV positive 2–3 years posttransplantation, and 1 was HEV positive >4 years after transplantation. Of the 15 organ donors, 14 were negative for HEV IgG and HEV IgM, and 1 was negative for HEV IgG and positive for HEV IgM. All 15 organ donors were negative for HEV RNA.

We determined how many of the 34 HEV-infected patients had required a blood transfusion during the 3-month period before onset of hepatitis or during the year before HEV seroconversion. Only 2 liver transplant recipients (17%) were given transfusions of concentrated erythrocytes (one 2 months before and one 3 weeks before infection with HEV was diagnosed). All 12 donors of these blood transfusions were negative for HEV RNA. Eleven of them were negative for HEV IgG and HEV IgM, and 1 was positive for HEV IgG and negative for HEV IgM.

### Description of HEV Case-Patients

We identified 34 patients with HEV infection (9 women and 25 men, mean ± SD age at diagnosis 46 ± 13 years). HEV genotypes for 21 patients were identified: 15 genotype 3f strains, 5 genotype 3c strains, and 1 genotype 3 strain with an undetermined subtype. Infections became chronic in 16 patients (47%), which was defined as HEV RNA in plasma for >6 months. None of the 18 patients who cleared virus after the acute phase showed any reactivation or reinfection during follow-up. Multivariate analysis showed that the independent risk factors associated with HEV infection (Table 3) were a younger age at transplantation (<52 years) and having a liver transplant.

**Table 3:** Analysis of risk factors for acquiring HEV infection in 601 solid-organ transplant recipients, France, January 2004–December 2008*

Characteristic	Univariate analysis			Multivariate analysis	
	Relative risk (95% CI)	p value		Relative risk (95% CI)	p value
Male sex	1.56 (0.73–3.35)	0.24		–	–
Age at transplantation <52 y	2.58 (1.20–5.53)	0.01		2.8 (1.3–6.0)	0.008
Living in rural area (<20,000 inhabitants)	0.6 (0.28–1.32)	0.21			
Having a liver transplant	1.78 (0.88–3.60)	0.10		2.03 (1–4.13)	0.05
Indication of liver transplantation					
Alcohol	0.95 (0.29–3.10)	0.94		–	–
Hepatitis B or C	1.34 (0.47–3.80)	0.58		–	–
Autoimmune	3.1 (0.75–13.15)	0.11		–	–
Other causes	2.52 (0.77–8.24)	0.12		–	–
Indication of kidney transplantation					
Glomerulonephritis	0.49 (0.19–1.28)	0.15		–	–
Genetic nephritis	1.06 (0.37–3.02)	0.90		–	–
Pyelonephritis or interstitial nephritis	0.20 (0.02–1.52)	0.13		–	–
Nephroangiosclerosis	1.45 (0.55–3.68)	0.46		–	–
Other causes	2.52 (0.77–8.24)	0.12		–	–
Induction therapy					
Yes	0.83 (0.39–1.74)	0.62		–	–
ATG	1.19 (0.53–2.65)	0.66		–	–
Anti-IL2R	0.95 (0.49–1.94)	0.95		–	–
Immunosuppressive therapy at discharge					
Belatacept	0.49 (0.06–3.60)	0.48		–	–
Cyclosporine A	0.72 (033–1.55)	0.41		–	–
Tacrolimus	1.49 (0.73–3.07)	0.27		–	–
Steroids	0.74 (0.17–3.11)	0.68		–	–
Mycophenolate	0.84 (0.34–2.02)	0.69		–	–
Azathioprine	0	1			
Double regimen	1.07 (0.50–2.30)	0.85		­–	–
Triple regimen	0.99 (0.46–2.13)	0.99			

*HEV, hepatitis E virus: CI, confidence interval; –, not significant; ATG, antithymoglobulin; anti-IL2R, interleukin-2 receptor blockers.

## Discussion

We followed up 700 patients who had received a kidney or liver transplant during 2004–2008. The incidence of de novo HEV infection was high (3.2 cases/100 person-years), but no infections were reactivated in this population.

The pretransplant HEV seroprevalence (14.7%) was similar to that of blood donors in the same area (16.6%) (*22*), but higher than in liver transplant recipients in the Netherlands (2.1%) (*28*), Germany (4%) (*29*), and Spain (2.7%) (*30*). Seven patients had HEV IgG and IgM at transplantation, but none had HEV RNA. These results suggest that HEV infection occurred shortly before transplantation. Ten patients were positive for HEV IgM but negative for HEV IgG and HEV RNA. None of the 10 patients showed delayed seroconversion for IgG during follow-up. Although the Adaltis EIAgen anti-HEV IgM Kit is highly specific (*31*), a false-positive result cannot be excluded. However, prolonged persistence of HEV IgM for >1 year after resolution of HEV infection has been reported (*32*). Because the anti-HEV IgG test we used could be relatively insensitive (*33**,**34*), absence of IgG seroconversion is compatible with a recent infection.

A recent study reported HEV reactivation in a patient with acute lymphoblastic leukemia after allogeneic stem cell transplantation (*21*). HEV may persist in hepatocytes or macrophages, as do other RNA viruses (*35**,**36*). We therefore investigated the possibility of reactivation in our patients who had antibodies against HEV but no HEV RNA at transplantation. We tested for HEV RNA at the first annual checkup and whether liver enzyme activities were increased during follow-up. We found no HEV infection in these patients. In addition, 32 (35.9%) of these patients no longer had HEV IgG after transplantation. The delay in disappearance of HEV IgG is poorly documented in immunocompetent or immunocompromised patients. The sensitivity of the IgG test may influence this delay, as recently documented by Bendall et al. in immunocompetent patients (*33*). This disappearance of antibodies against HEV may be caused by immunosuppressive treatment. Mycophenolic acid and rapamycin can decrease antibody synthesis (*37**,**38*), but we found no association between use of mycophenolic acid and loss of antibodies against HEV.

The incidence of HEV infection after transplantation was high; we identified 34 patients with HEV infection. Most patients were infected with HEV genotype 3f, which is the most prevalent genotype in the study area (*26*). Incidence rates for each year after transplantation were similar. Likewise, incidence of HEV infection in SOT patients did not vary according to year of transplantation. This finding is consistent with that of a study in immunocompetent patients in the same area, in which the frequency of HEV infections did not vary over time (*24*). No studies have investigated the incidence of HEV in immunocompromised patients. The prevalence of acquired HEV after liver transplantation in the Netherlands was only 1% (*28*). This discrepancy with our data reinforces the need for studies on factors associated with HEV infection in different regions of Europe.

We investigated HEV transmission by organ transplant by detection of antibodies against HEV and HEV RNA in the blood of donors who provided organs to 15 patients infected with HEV. No HEV RNA was detected in blood samples of organ donors; thus, they were considered to be noninfectious. Because transfusion-associated hepatitis E has been described in Europe (*9**,**10*), we assayed virologic markers in frozen blood samples of 12 blood donors involved in transfusions for 2 HEV-infected recipients, but none of the samples were positive.

Multivariate analysis identified an age <52 years as a factor associated with HEV infection in SOT patients. A previous report found that a younger age was associated with primary cytomegalovirus infection in liver transplant recipients (*39*). However, the mechanisms underlying these observations for cytomegalovirus and HEV infections are not clear. HEV infections are more frequent in older persons in industrialized countries (*2**,**24*). Being a liver transplant recipient is another risk factor for acquiring HEV infection. Local inflammation may cause liver transplant patients to have a greater risk for HEV infection.

Immunocompromised patients have a higher risk for chronic infection with HEV, which may lead to cirrhosis (*15**,**23*). The high incidence of HEV infections in the study area and the fact that no vaccine against HEV is available make it essential to identify routes of transmission so that preventive measures can be taken. A case–control study showed that factors associated with HEV infection in immunocompromised patients in the study area were the consumption of game meat, food products made with pork, and mussels (*32*). Thorough cooking of game meat and pork products and better information on HEV transmission would help minimize the risk for HEV infection.

Our study had several limitations. We investigated HEV reactivation at the first annual checkup and in patients with hepatic cytolysis. Thus, we may have missed asymptomatic reactivation that occurred during the rest of the follow-up period. In addition, 102 patients who were HEV negative at transplantation were lost to follow-up, which may have affected the incidence of HEV cases. The de novo cases were identified by detection of HEV RNA or HEV IgG. Low sensitivity of the HEV IgG assay may have also influenced incidence of HEV infection. Lastly, we screened only 44% of those who donated an organ to the HEV-infected patients and may have missed a transplant-associated infection.

In conclusion, we detected a high incidence of HEV infection in SOT patients in southern France. Liver transplant recipients may be at greater risk for HEV infection than kidney transplant recipients. Patients on a transplant waiting list could be vaccinated when a suitable vaccine becomes available. Until then, the high risk for HEV infection becoming chronic indicates that the diets of SOT patients should be closely monitored.

## References

[1] Emerson SU, Purcell RH. Hepatitis E virus. Rev Med Virol. 2003;13:145–54. 10.1002/rmv.38412740830

[2] Dalton HR, Bendall R, Ijaz S, Banks M. Hepatitis E: an emerging infection in developed countries. Lancet Infect Dis. 2008;8:698–709. 10.1016/S1473-3099(08)70255-X18992406

[3] Meng XJ. Recent advances in hepatitis E virus. J Viral Hepat. 2010;17:153–61. 10.1111/j.1365-2893.2009.01257.x20040046

[4] Christensen PB, Engle RE, Hjort C, Homburg KM, Vach W, Georgsen J, Time trend of the prevalence of hepatitis E antibodies among farmers and blood donors: a potential zoonosis in Denmark. Clin Infect Dis. 2008;47:1026–31. 10.1086/59197018781880PMC2803052

[5] Meng XJ, Wiseman B, Elvinger F, Guenette DK, Toth TE, Engle RE, Prevalence of antibodies to hepatitis E virus in veterinarians working with swine and in normal blood donors in the United States and other countries. J Clin Microbiol. 2002;40:117–22. 10.1128/JCM.40.1.117-122.200211773103PMC120098

[6] Li TC, Chijiwa K, Sera N, Ishibashi T, Etoh Y, Shinohara Y, Hepatitis E virus transmission from wild boar meat. Emerg Infect Dis. 2005;11:1958–60.1648549010.3201/eid1112.051041PMC3367655

[7] Tei S, Kitajima N, Takahashi K, Mishiro S. Zoonotic transmission of hepatitis E virus from deer to human beings. Lancet. 2003;362:371–3. 10.1016/S0140-6736(03)14025-112907011

[8] Yazaki Y, Mizuo H, Takahashi M, Nishizawa T, Sasaki N, Gotanda Y, Sporadic acute or fulminant hepatitis E in Hokkaido, Japan, may be food-borne, as suggested by the presence of hepatitis E virus in pig liver as food. J Gen Virol. 2003;84:2351–7. 10.1099/vir.0.19242-012917455

[9] Boxall E, Herborn A, Kochethu G, Pratt G, Adams D, Ijaz S, Transfusion-transmitted hepatitis E in a 'nonhyperendemic' country. Transfus Med. 2006;16:79–83. 10.1111/j.1365-3148.2006.00652.x16623913

[10] Colson P, Coze C, Gallian P, Henry M, De Micco P, Tamalet C. Transfusion-associated hepatitis E, France. Emerg Infect Dis. 2007;13:648–9. 10.3201/eid1304.06138717561564PMC2725983

[11] Mansuy JM, Huynh A, Abravanel F, Recher C, Peron JM, Izopet J. Molecular evidence of patient-to-patient transmission of hepatitis E virus in a hematology ward. Clin Infect Dis. 2009;48:373–4. 10.1086/59589319128164

[12] Dalton HR, Thurairajah PH, Fellows HJ, Hussaini HS, Mitchell J, Bendall R, Autochthonous hepatitis E in southwest England. J Viral Hepat. 2007;14:304–9. 10.1111/j.1365-2893.2006.00800.x17439519

[13] Péron JM, Bureau C, Poirson H, Mansuy JM, Alric L, Selves J, Fulminant liver failure from acute autochthonous hepatitis E in France: description of seven patients with acute hepatitis E and encephalopathy. J Viral Hepat. 2007;14:298–303. 10.1111/j.1365-2893.2007.00858.x17439518

[14] Gérolami R, Moal V, Picard C, Colson P. Hepatitis E virus as an emerging cause of chronic liver disease in organ transplant recipients. J Hepatol. 2009;50:622–4. 10.1016/j.jhep.2008.12.00819157619

[15] Haagsma EB, van den Berg AP, Porte RJ, Benne CA, Vennema H, Reimerink JH, Chronic hepatitis E virus infection in liver transplant recipients. Liver Transpl. 2008;14:547–53. 10.1002/lt.2148018383084

[16] Kamar N, Selves J, Mansuy JM, Ouezzani L, Peron JM, Guitard J, Hepatitis E virus and chronic hepatitis in organ-transplant recipients. N Engl J Med. 2008;358:811–7. 10.1056/NEJMoa070699218287603

[17] Ollier L, Tieulie N, Sanderson F, Heudier P, Giordanengo V, Fuzibet JG, Chronic hepatitis after hepatitis E virus infection in a patient with non-Hodgkin lymphoma taking rituximab. Ann Intern Med. 2009;150:430–1.1929308410.7326/0003-4819-150-6-200903170-00026

[18] Péron JM, Mansuy JM, Recher C, Bureau C, Poirson H, Alric L, Prolonged hepatitis E in an immunocompromised patient. J Gastroenterol Hepatol. 2006;21:1223–4. 10.1111/j.1440-1746.2006.04209.x16824086

[19] Colson P, Kaba M, Moreau J, Brouqui P. Hepatitis E in an HIV-infected patient. J Clin Virol. 2009;45:269–71. 10.1016/j.jcv.2009.06.00219757504

[20] Dalton HR, Bendall RP, Keane FE, Tedder RS, Ijaz S. Persistent carriage of hepatitis E virus in patients with HIV infection. N Engl J Med. 2009;361:1025–7. 10.1056/NEJMc090377819726781

[21] le Coutre P, Meisel H, Hofmann J, Rocken C, Vuong GL, Neuburger S, Reactivation of hepatitis E infection in a patient with acute lymphoblastic leukaemia after allogeneic stem cell transplantation. Gut. 2009;58:699–702. 10.1136/gut.2008.16557119359434

[22] Mansuy JM, Legrand-Abravanel F, Calot JP, Peron JM, Alric L, Agudo S, High prevalence of anti-hepatitis E virus antibodies in blood donors from south west France. J Med Virol. 2008;80:289–93. 10.1002/jmv.2105618098159

[23] Kamar N, Abravanel F, Selves J, Garrouste C, Esposito L, Lavayssiere L, Influence of immunosuppressive therapy on the natural history of genotype 3 hepatitis-E virus infection after organ transplantation. Transplantation. 2010;89:353–60. 10.1097/TP.0b013e3181c4096c20145528

[24] Mansuy JM, Abravanel F, Miedouge M, Mengelle C, Merviel C, Dubois M, Acute hepatitis E in south-west France over a 5-year period. J Clin Virol. 2009;44:74–7. 10.1016/j.jcv.2008.09.01018993112

[25] Mansuy JM, Peron JM, Abravanel F, Poirson H, Dubois M, Miedouge M, Hepatitis E in the south west of France in individuals who have never visited an endemic area. J Med Virol. 2004;74:419–24. 10.1002/jmv.2020615368508

[26] Legrand-Abravanel F, Mansuy JM, Dubois M, Kamar N, Peron JM, Rostaing L, Hepatitis E virus genotype 3 diversity, France. Emerg Infect Dis. 2009;15:110–4. 10.3201/eid1501.08029619116067PMC2660688

[27] Lu L, Li C, Hagedorn CH. Phylogenetic analysis of global hepatitis E virus sequences: genetic diversity, subtypes and zoonosis. Rev Med Virol. 2006;16:5–36. 10.1002/rmv.48216175650

[28] Haagsma EB, Niesters HG, van den Berg AP, Riezebos-Brilman A, Porte RJ, Vennema H, Prevalence of hepatitis E virus infection in liver transplant recipients. Liver Transpl. 2009;15:1225–8. 10.1002/lt.2181919790147

[29] Pischke S, Suneetha PV, Baechlein C, Barg-Hock H, Heim A, Kamar N, Hepatitis E virus infection as a cause of graft hepatitis in liver transplant recipients. Liver Transpl. 2010;16:74–82. 10.1002/lt.2195819866448

[30] Buti M, Cabrera C, Jardi R, Castells L, Esteban R. Are recipients of solid organ transplantation a high-risk population for hepatitis E virus infection? Liver Transpl. 2010;16:106–7, author reply 108. 10.1002/lt.2192520035519

[31] Legrand-Abravanel F, Thevenet I, Mansuy JM, Saune K, Vischi F, Peron JM, Good performance of immunoglobulin M assays in diagnosing genotype 3 hepatitis E virus infections. Clin Vaccine Immunol. 2009;16:772–4. 10.1128/CVI.00438-0819321696PMC2681577

[32] Legrand-Abravanel F, Kamar N, Sandres-Saune K, Garrouste C, Dubois M, Mansuy JM, Characteristics of autochthonous hepatitis E virus infection in solid-organ transplant recipients in France. J Infect Dis. 2010;202:835–44. 10.1086/65589920695798

[33] Bendall R, Ellis V, Ijaz S, Ali R, Dalton H. A comparison of two commercially available anti-HEV IgG kits and a re-evaluation of anti-HEV IgG seroprevalence data in developed countries. J Med Virol. 2010;82:799–805. 10.1002/jmv.2165620336757

[34] Mast EE, Alter MJ, Holland PV, Purcell RH. Evaluation of assays for antibody to hepatitis E virus by a serum panel. Hepatitis E Virus Antibody Serum Panel Evaluation Group. Hepatology. 1998;27:857–61. 10.1002/hep.5102703319500718

[35] Labadie K, Larcher T, Joubert C, Mannioui A, Delache B, Brochard P, Chikungunya disease in nonhuman primates involves long-term viral persistence in macrophages. J Clin Invest. 2010;120:894–906. 10.1172/JCI4010420179353PMC2827953

[36] Way SJ, Lidbury BA, Banyer JL. Persistent Ross River virus infection of murine macrophages: an in vitro model for the study of viral relapse and immune modulation during long-term infection. Virology. 2002;301:281–92. 10.1006/viro.2002.158712359430

[37] Luo H, Chen H, Daloze P, Chang JY, St-Louis G, Wu J. Inhibition of in vitro immunoglobulin production by rapamycin. Transplantation. 1992;53:1071–6. 10.1097/00007890-199205000-000191585470

[38] Rentenaar RJ, van Diepen FN, Meijer RT, Surachno S, Wilmink JM, Schellekens PT, Immune responsiveness in renal transplant recipients: mycophenolic acid severely depresses humoral immunity in vivo. Kidney Int. 2002;62:319–28. 10.1046/j.1523-1755.2002.00425.x12081594

[39] Arthurs SK, Eid AJ, Pedersen RA, Dierkhising RA, Kremers WK, Patel R, Delayed-onset primary cytomegalovirus disease after liver transplantation. Liver Transpl. 2007;13:1703–9. 10.1002/lt.2128018044717

# Research article: Hepatitis E Virus Infection without Reactivation in Solid-Organ Transplant Recipients, France

**DOI:** 10.3201/eid1701.100527

**Authors:** Florence Legrand-Abravanel, Nassim Kamar, Karine Sandres-Saune, Sebastien Lhomme, Jean-Michel Mansuy, Fabrice Muscari, Federico Sallusto, Lionel Rostaing, Jacques Izopet

**Affiliations:** Author affiliations: Institut National de la Santé et de la Recherche Médicale, Toulouse, France (F. Legrand-Abravanel, N. Kamar, K. Sandres-Saune, S. Lhomme, L. Rostaing, J. Izopet);; Centre Hospitalier Universitaire Toulouse, Toulouse (F. Legrand-Abravanel, N. Kamar, K. Sandres-Saune, S. Lhomme, J.-M. Mansuy, F. Muscari, F. Sallusto, L. Rostaing, J. Izopet)

## Earning CME Credit

To obtain credit, you should first read the journal article. After reading the article, you should be able to answer the following, related, multiple-choice questions. To complete the questions and earn continuing medical education (CME) credit, please go to www.medscapecme.com/journal/eid. Credit cannot be obtained for tests completed on paper, although you may use the worksheet below to keep a record of your answers. You must be a registered user on Medscape.com. If you are not registered on Medscape.com, please click on the New Users: Free Registration link on the left hand side of the website to register. Only one answer is correct for each question. Once you successfully answer all post-test questions you will be able to view and/or print your certificate. For questions regarding the content of this activity, contact the accredited provider, CME@medscape.net. For technical assistance, contact CME@webmd.net. American Medical Association’s Physician’s Recognition Award (AMA PRA) credits are accepted in the US as evidence of participation in CME activities. For further information on this award, please refer to http://www.ama-assn.org/ama/pub/category/2922.html. The AMA has determined that physicians not licensed in the US who participate in this CME activity are eligible for *AMA PRA Category 1 Credits*™. Through agreements that the AMA has made with agencies in some countries, AMA PRA credit is acceptable as evidence of participation in CME activities. If you are not licensed in the US and want to obtain an AMA PRA CME credit, please complete the questions online, print the certificate and present it to your national medical association.

## Article Title: Hepatitis E Virus Infection without Reactivation in Solid-Organ Transplant Recipients, France

## CME Questions

**1. Based on results from this study, which of the following patients would be anticipated to be at highest risk for acquisition of hepatitis E virus (HEV) infection?**A. A 65-year-old man undergoing kidney transplantationB. A 45-year-old woman undergoing liver transplantationC. A 76-year-old man 1 year post liver transplant.D. A 36-year-old woman 6 months post kidney transplant**2. A 49-year-old female patient who is HIV positive undergoes a liver transplant. Six months later she is found to be HEV positive. Which of the following factors would increase her risk to evolve to a chronic infection?**A. Alcohol useB. Female sexC. Immunocompromise associated with HIV infectionD. Comorbid hepatitis C virus or hepatitis B virus infection

## Activity Evaluation

**Table:** 

1. The activity supported the learning objectives.				
Strongly Disagree				Strongly Agree
1	2	3	4	5
2. The material was organized clearly for learning to occur.				
Strongly Disagree				Strongly Agree
1	2	3	4	5
3. The content learned from this activity will impact my practice.				
Strongly Disagree				Strongly Agree
1	2	3	4	5
4. The activity was presented objectively and free of commercial bias.				
Strongly Disagree				Strongly Agree
1	2	3	4	5
